# Quantification of Fetal Renal Function Using Fetal Urine Production Rate and Its Reflection on the Amniotic and Fetal Creatinine Levels During Pregnancy

**DOI:** 10.3389/fped.2022.841495

**Published:** 2022-03-03

**Authors:** Udoamaka Ezuruike, Alexander Blenkinsop, Amita Pansari, Khaled Abduljalil

**Affiliations:** Certara UK Limited (Simcyp Division), Sheffield, United Kingdom

**Keywords:** fetus, pregnancy, urine production, renal function, PBPK, creatine, GFR

## Abstract

Adequate prediction of fetal exposure of drugs excreted by the kidney requires the incorporation of time-varying renal function parameters into a pharmacokinetic model. Published data on measurements of fetal urinary production rate (FUPR) and creatinine at various gestational ages were collected and integrated for prediction of the fetal glomerular filtration rate (GFR). The predicted GFR values were then compared to neonatal values recorded at birth. Collected data for FUPR across different gestational ages using both 3D (*N* = 517) and 2D (*N* = 845) ultrasound methods showed that 2D techniques yield significantly lower estimates of FUPR than 3D (*p* < 0.0001). A power law function was shown to best capture the change in FUPR with fetal age (FA) for both 2D ($FUPR_{2D}\left(\frac{mL}{\min}\right)=0.000169\;{\;\text{FA}}^{2.19}$); and 3D ($FUPR_{3D}\;\left(\frac{mL}{\min}\right)=\;3.21\times 10^{-7}\;{\text{FA}}^{4.21}$) data. The predicted FUPR based on the observed 3D data was shown to be strongly linearly related (*R*^2^ = 0.95) to measured values of amniotic creatinine concentration (*N* = 664). The FUPR_3D_ data together with creatinine levels in the fetal urine and serum resulted in median predicted fetal GFR values of 0.47, 1.2, 2.5, and 4.9 ml/min at 23, 28, 33, and 38 weeks of fetal age (50% CV), respectively. These values are in good agreement with neonatal values observed immediately at birth. The derived FUPR and creatinine functions can be utilized to assess fetal renal maturation and predict fetal renal clearance.

## Introduction

The kidney is the main organ responsible for urine formation and excretion of drugs from the body. The kidneys develop between the 5th and 12th week of fetal life and increase significantly during the second half of pregnancy, owing to intense nephrogenesis (nephron formation) and a significant increase in the number of nephrons (1). Previous meta-analysis showed that the average weight of both kidneys increased from 0.22 g at 12 GA (gestational age in weeks) to 15.9, and 27.3 g at 32, and 40 GA, respectively (2). By the 13th week, the kidneys start to produce urine, due largely to the increase in the number of nephrons, which is a direct indicator of the functional capacity of the kidney. Nnephrogenesis commences at around 8 weeks GA. By 20 weeks, about 30% of adult number of nephrons have been formed and by birth in a term born infant, nephrogenesis is complete. This indicates that ~60% of nephrons are formed in the second half of gestation (3, 4). The number of nephrons in the normal human kidney varies from ~250,000 to over two million (5).

Postnatal renal function can be quantified by many different methods. However, limited options are available for assessing fetal renal function. The options available include amniotic fluid volume, ultrasonographic appearance of kidneys, urine production and the biochemical composition of fetal urine (6). Although the details of fetal kidney physiology are well-described, the clinical evaluation of fetal glomerular filtration rate (GFR) remains a challenging exercise. GFR either directly measured based on the clearance of exogenous filtration markers such as inulin; or estimated from the clearance of endogenous filtration markers-creatinine and cystatin C is regarded as the mainstay in clinical practice for assessing postnatal renal function (7), albeit with a number of identified limitations for each of the methods (8, 9).

Due to clinical and ethical constraints, few studies have reported GFR values, either from pre-clinical species (10–12) or from infants after birth, which are based on inulin clearance (13, 14) or creatinine clearance (15, 16), and are used in lieu of human fetal GFR at different gestational ages. It is known however, that the transition from fetal to new-born life is accompanied by multifactorial hemodynamic and functional changes. Fetal kidneys receive about 5% of fetal cardiac output (17). Postnatal kidneys on the other hand, received about 16% of cardiac output in a 2-days old new-born infant born at 35.4 GAs (18). Therefore, postnatal estimation of new-born GFR is likely to differ from the fetal GFR at term. Since these physiological parameters are gestational-age dependent, the neonatal GFR immediately at birth will also be gestational age dependent (9).

Measuring fetal GFR has its place in pharmacokinetics (PK) for predicting the fetal renal clearance of xenobiotics and their level in the amniotic fluid. Determination of fetal GFR requires quantifications of its variables, including urine flow, and concentration of the markers in the fetus, and in the fetal urine at different gestational weeks. Since inulin cannot be injected to the fetus for measuring GFR, the use of endogenous creatinine or Cystatin C is an alternative method.

The first direct measurement of fetal urinary production/flow rate was demonstrated by Campbell et al. (19). The measurement was based on volume estimations of the bladder, using static measurements of its longitudinal and transverse sections and regular observations of its emptying and filling, using 2-dimentional (2D) ultrasound (19). Since then, advancements in real time ultrasound measurements, including the use of 3D ultrasound with integrated “Virtual Organ Computer-aided AnaLysis” (VOCAL) software have resulted in more accurate visualizations of the filling and emptying cycles of the fetal bladder (20). The hourly fetal urine production rate (FUPR) can then be estimated, either by regression analysis of the acquired bladder volumes at different time points within the filling phase, or by the difference between the maximum and minimum bladder volumes divided by the time interval.

The aim of this paper was to assess the fetal GFR *via* undertaking a thorough analysis of the published literature on FUPR and fetal creatinine concentration in the fetal serum (SerCr), urine (UrCr), and in the amniotic fluid during fetal development.

## Methods

### Literature Search

Searches for published literature articles relating to fetal urinary flow rate were carried out using PubMed (https://pubmed.ncbi.nlm.nih.gov/) and Google Scholar (https://scholar.google.com/) in April 2021. The key words utilized in the searches include “fetal urine/urinary production”, “fetal urine/urinary flow”, “fetal glomerular filtration” and “fetal GFR”. An additional search was carried out to identify published articles with measured values of creatinine in the fetal urine, serum and amniotic compartments at different gestational ages using for example the following search terms for creatinine- “amniotic fluid constituents”, “amniotic fluid creatinine”, “fetal amniotic creatinine”, “fetal urine creatinine”, “fetal urine biochemistry”, and “fetal urinary creatinine”. References within the individual search results were also reviewed to identify any additional articles that may have been missed in the initial searches.

Studies from both searches whose data were retained for the analysis were those that met the following criteria- (1) the subjects were healthy females with no known pregnancy-related complications, (2) the pregnancy was described as normal, and were not characterized by any known fetal abnormalities, including kidney abnormalities (3) only singleton pregnancies and (4) an explicit mention of gestational age at which fetal urine production rate or creatinine concentration was measured. Control data from subjects meeting the above-mentioned criteria for studies that were conducted specifically to look at complicated pregnancies were also included. One of the initial selection criteria was to only include studies carried out in a Caucasian population. However, due to the paucity of published data where only Caucasian population were considered, as well as evidence from the data suggesting there are no significant ethnic differences (Table 1), this was removed from the inclusion criteria.

**Table 1:** Summary of studies with measured values of fetal urine flow rates (ml/h) at different gestational ages in normal pregnancy.

**Reference and country of study**	**Number of subjects (Status)**	**Gestational weeks (range)**	**FUPR (Mean ± SD)**	**Method of measurement (calculation formula)**	**Relationship between FUPR and GA (weeks)**
Campbell et al. (19) (UK)	50 (Normal pregnancy)	32 34 35 37 38 40	12.2 ± 1.5 14.5 ± 1.0 17.4 ± 2.2 20.8 ± 2.6 22.7 ± 2.9 28.2 ± 1.7	2D static ultrasound (Ovoid formula) Bladder volume was observed at 15 to 30 min interval	Linear, HFUPR = −45.3 + 1.8 GA, R = 0.8955
Wladimiroff and Campbell (21) (UK)	92 (Normal pregnancy)	30 32 34 36 38 40	9.6 ± 0.9 12.2 ± 1. 4 14.9 ± 1.3 18.3 ± 2.4 24.1 ± 3.2 27.3 ± 2.3	2D static ultrasound (Ovoid formula) Bladder volume was observed at 15 to 30 min intervals	Linear, HFUPR = −47.71 + 1.87 GA, R = 0.922
Kurjak et al. (22) (Finland)	255 (Normal pregnancy)	22–26 27–31 32–36 37–41	2.2–5.7 6.7–10.8 11.8–19.0 20.9–26.7	2D static ultrasound (Ovoid formula) Bladder volume was observed at 15 to 30 min intervals	No derived function but a linear relationship suggested.
Rabinowitz et al. (23) (UK)	85 (Normal pregnancy)	20–28 30–34 36–40	5–14 18–27 33–51	2D real-time ultrasound (Ovoid formula) Bladder volume was observed at 2 to 5 min intervals	Log linear Log_10_ (HFUPR + 3) = 0.088 + 0.041 GA
Deutinger et al. (24) (Austria)	52 (Normal pregnancy)	28–30 31–35 36–40	5.2–11.2 6.3–18.1 12.1–28.9	2D ultrasound (Ovoid formula)	None but linear relationship proposed.
Shin et al. (25) (Japan)	187 (Normal pregnancy)	20 28 35 40	1.74 11.40 32.40 34.80	2D real-time ultrasound Bladder volume was observed at 2 to 10 min intervals (Ovoid formula)	No derived function. Marked increase in HFUPR up to 38 GAs followed by a decrease.
Fagerquist et al. (26) (Sweden)	62 (Small-, large- and heavy-for-gestational age)	20 25 30 35 40	4.2 12.1 22.7 36.1 52.2	2D ultrasound (Ovoid formula)	2nd order Polynomial, HFUPR = −0.258430 – 0.865381 GA + 0.054410 GA^2^
Lee et al. (27) (South Korea)^*^	154 (Normal pregnancy)	24–29 30–35 36–40	7.3–16.6 23.9–44.2 57.2–71.4	Linear regression analysis of bladder volume changes measured using 3D ultrasound imaging and VOCAL analysis (30° rotational angle)	2nd order Polynomial, Ln (HUPR) = −6.29582 + 0.43924 GA + 0.000432 GA^2^
Touboul et al. (20) (France)^*^	167 (Normal pregnancy)	20–28 29–34 35–40	1.9–14.7 18.1–47.2 56.1–125.2	Regression analysis of bladder volume changes with time acquired using 3D ultrasound with VOCAL software (30° rotational angle)	Power, HFUPR = 3E – 08 × GA^6.005^
Stigter et al. (28) (The Netherlands)	95 (Normal pregnancy)	37–41		Regression analysis of bladder volume measured using real time ultrasonography and estimated using the mathematical average of the sagittal and coronal area.	Not directly relating HFUPR to GA
Maged et al. (29) (Egypt)	100 (Normal pregnancy)	25 30 35 40	12.3 14.4 56.1 90.7	Change in bladder volume with time measured using 3D VOCAL.	Linear regression model proposed but no estimated function derived.
Lee et al. (30) (South Korea)	141 (Normal pregnancy)	37	49 ± 32.6	Same as Lee (27)	None as measurement at only 37W (GA)

* *Median*.

### Data Analysis

From each individual study, the mean and standard deviation (SD) for both the fetal urinary production rate and the concentration of creatinine in the amniotic fluid, urine and fetal serum at the different gestational ages in weeks (GA) were calculated. The fetal age in weeks (FA) was calculated from the GA (FA= GA-2). The weighted mean (μ) from the different studies was then calculated using Equation 1:


(1) $$\begin{array}{l} \mu =\;\frac{\sum_{j=1}^{N}n_{j}.x_{j}}{\sum_{j=1}^{N}n_{j}} \end{array}$$

where *n*_*j*_ is the number of observations in the *j*^*th*^ study, *x*_*j*_ is the mean value from the *j*^*th*^ study and *N* is the total number of studies for the current fetal age.

The weighted standard deviation (σ^*^) was calculated using Equation 2:


(2) $$\begin{array}{l} \sigma^{*}=\sqrt{\frac{\sum_{j=1}^{N}n_{j}\left(x_{j}^{2}+\sigma_{j}^{2}\right)-\mu^{2}\sum_{j=1}^{N}n_{j}}{\sum_{j=1}^{N}n_{j}}} \end{array}$$

Where *n*_*j*_ is the number of observations in the *j*^*th*^ study; *N* is the number of studies for the current fetal age; *x*_*j*_ and σ_*j*_ are the mean and standard deviation of the FUPR respectively in the *j*^*th*^ study, and μ is the weighted average across all *N* studies for the current fetal age (as given by Equation 1).

To quantify the fit to data of the regression analyses, a weighted mean squared error (MSE) was used, given by Equation 3:


(3) $$\begin{array}{l} MSE=\frac{1}{W.\sum_{i=1}^{W}n_{i}}\sum_{i=1}^{W}n_{i}{\left(y_{i}-u_{i}\right)}^{2} \end{array}$$

Where *W* is the total number of sampling times for the different fetal ages; *n*_*i*_ is the total number of observations across all studies in the *i*^*th*^ week; *y*_*i*_ is the model predicted FUPR in the *i*^*th*^ week, and μ_*i*_ is the weighted mean of the FUPR of all subjects in the *i*^*th*^ week across all studies.

To identify which function best fits the data, weighted regression analyses was carried out for different growth functions including, linear, exponential, polynomial, and power law. Each function's parameters were optimized using the Microsoft built-in Excel's LINEST function. The weighted mean squared error and coefficient of determination (*R*^2^) value was then calculated for each of the three functions.

### Calculation of Fetal GFR

The fetal creatinine based GFR was calculated according to Equation 4:


(4) $$\begin{array}{r} Fetal\;GFR\;\left(mL/\min\right)\\ =\frac{FUPR\;\left(mL/\min\right)\;*Fetal\;UrCr\;\left(mg/L\right)}{Fetal\;SerCr\;\left(mg/L\right)} \end{array}$$

To account for interindividual variability, a sample of 800 fetuses was generated and data at six gestational ages (15, 20, 25, 30, 35, and 40 weeks) were summarized for the changes in the parameter values using constant coefficient of variations (CV), based on the observed data, of 30% for FUPR, 25% for UrCr, and 25% for SerCr. Due to the absence of fetal GFR measurements, data from preterm and term neonates observed immediately after birth were compared against the predicted fetal GFR using Equation 4.

## Results

Thirteen different original research articles, which altogether provided over 1,300 measured values of FUPR from pregnant women across different gestational ages and met the pre-defined criteria, were identified in the literature (Table 1). The FUPR was highly variable in the collated data even for the same gestational age due to the measurement technique used. Seven of these studies, carried out between 1973 and 2001 used 2D ultrasonographic techniques, either with static or real-time measurements, with a total of 845 measurements. The other five studies conducted between 2007 and 2014 utilized 3D ultrasound with integrated VOCAL software with a total of 517 measurements.

The FUPR from these studies ranged from a minimum reported value of 0.5 ml/hr at 19 weeks GA (25), to 118.5 ml/hr at 40 weeks GA by Rabinowitz et al. (23), for the 2D ultrasound measurements; and from a minimum reported value of 3.25 ml/hr at 23 weeks GA to 125.2 ml/hr at 40 weeks GA (20), measured using the 3D VOCAL system. For most of the studies, the measured FUPR increased with gestational age, with further derivation of either a linear, exponential, or power function in some of the studies to describe the relationship between FUPR and GA (Table 1). The only exception was the study carried out by Shin et al., in which the FUPR seemed to decrease after 38 weeks GA (25).

**Figure 1:**
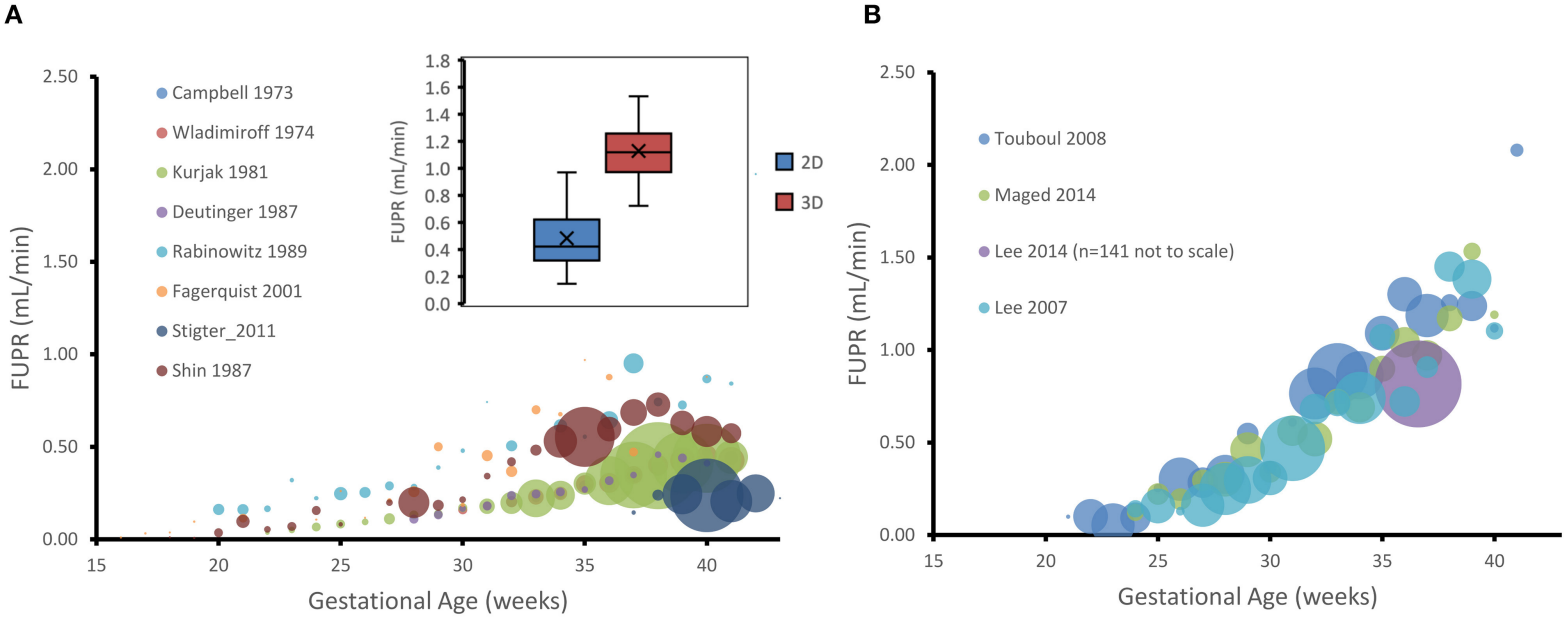
Gestational age plotted against hourly fetal urine production rate (FUPR) as measured either by 2D **(A)** or 3D **(B)** ultrasonic technique. Each data point is the mean FUPR from the subjects detailed in the studies shown in the legend for each age. The number of subjects averaged for each point are represented by the width of the bubble, ranging from, in A, *n* = 1 (26) to *n* = 39 (22) at week 38, and, in B to *n* = 14 (20) at week 33. Inset shows the data for estimates of FUPR calculated from either the 2D or 3D ultrasound techniques from 35 gestational weeks to term. Boxes: interquartile range. Median: midline. Crosses: mean. Whiskers: maximum and minimum.

A plot of the measured FUPR in ml/min against GA (weeks) from the different studies is shown in Figure 1, separated into data gathered using 2D (plot A) and those that used 3D (plot B) ultrasonic techniques. These plots show that the 2D technique yields estimates of urine production rates that are significantly less than measurements done using 3D ultrasound. To confirm this observation, the data was subset to isolate the mean production rates from 35 gestational weeks to term for both the 2D and 3D measurements. A two-tail, two-sample *t*-test assuming unequal variances, with alpha = 0.05, was carried out. Data from the 44 data points using 2D measurements (Mean = 0.48, SD = 0.21) and the 20 data points using 3D measurements (Mean = 1.18, SD = 0.30) from 35 to 40 GAs were significantly different (*p* < 0.0001).

### Prediction of FUPR From Fetal Age

**Figure 2:**
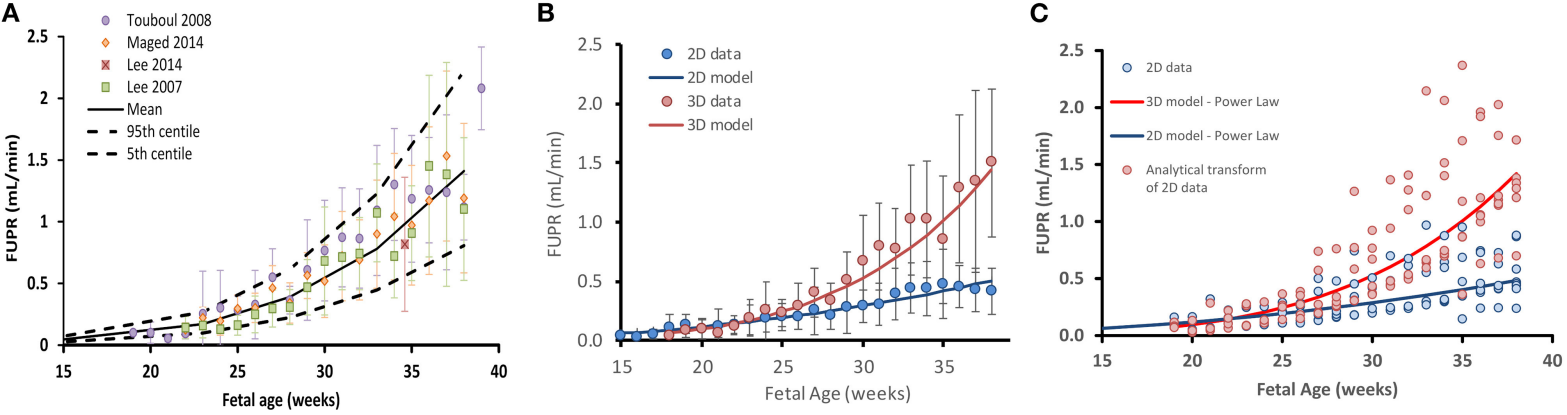
Fetal urine production rate (FUPR) during development. **(A)** FUPR as a function of fetal age (weeks). Colored filled circles are mean observations from 3D ultrasound data and error bars represent the standard deviation (SD). Solid black lines are predicted mean FUPR (From power law regression model, Equation 4) and dashed black lines represent the 5th and 95th percentiles for the observed data. **(B)** Observed mean 3D (red) and 2D (blue) ultrasound data. Each point is a weighted mean and standard deviation (bars), weighted by the number of subjects at each week. Power law regression models for each methodology are shown in solid lines. **(C)** Effect of transforming FUPR, as measured by 2D ultrasound (blue open circles). Summary data for every study plotted separately rather than averaged (as in the case in **B**). Resultant transformed data points (red open circles using Equation 7). Blue solid line: 2D power law regression model (Equation 5). Red solid line: 3D power law regression model (Equation 4).

The weighted mean for the collated data measured by 3D ultrasound indicated that the FUPR (mean ± SD) increases from 0.10 ± 0.08 ml/min at 21 GAs to 0.33 ± 0.15 ml/min at 30 GAs, reaching about 1.39 ± 0.64 ml/min at term. Since the 3D ultrasound data gives more accurate measurements of fetal bladder volume (31), the 3D data was used to create the predictive function for FUPR (Figure 2A). Results of FUPR data analysis for 3D studies indicated that the polynomial function provided the best fits to the observed data, however it predicted negative values at the beginning of the second trimester (Supplementary Figure 1A). In contrast, the power law function does not have this shortcoming and produced realistic FUPR estimates at all fetal ages, therefore, this power law function was selected (diagnostic statistics of the tested functions are available in the Supplementary Table 1).


(5) $$\begin{array}{l} FUPR_{3D}\;\left(\frac{mL}{\min}\right)=\;3.21\times 10^{-7}\;{FA}^{4.21} \end{array}$$

Where FA is fetal age in weeks.

The weighted mean for the collated data measured by 2D ultrasound indicated that the FUPR (mean ± SD) increases from 0.12 ± 0.1 ml/min at 21 GAs to 0.21 ± 0.11 ml/min at 30 GAs, reaching about 0.42 ± 0.19 ml/min at term. A power law function was selected among the tested functions (Supplementary Table 2), for the same reasons mentioned earlier for fitting 3D, resulting in Equation 6:


(6) $$\begin{array}{l} FUPR_{2D}\left(\frac{mL}{\min}\right)=1.69\times 10^{-4}\;{\;FA}^{2.19} \end{array}$$

Performance of these functions together with the data are shown in Figure 2B.

Since 2D ultrasound methods are more commonly used for fetal assessments, it would be useful to have a function that can transform 2D-derived estimates of fetal urine production rates into values that would have been obtained using the 3D technique. To this end, we derived the following analytical function by dividing the 3D model by the 2D model as shown in Equation 7.


(7) $$\begin{array}{l} FUPR_{3D}=1.89\times 10^{-3}FA^{2.02}FUPR_{2D} \end{array}$$

Where FUPR_2D is the estimate of FUPR as calculated using the 2D method and FUPR_3D is an estimate of fetal urine production rate that would have been obtained if the 3D technique had been employed instead. This equation can be applied as a correction function to transform 2D measurements to 3D estimates. The effect of applying this scaling function on the 2D data is shown in Figure 2C.

### Amniotic Creatinine Level

Sixteen different original research articles with measured values of creatinine concentrations in the amniotic fluid (*N* = 1,319) across various gestational ages, meeting the pre-defined inclusion criteria were identified (Table 2). The collected data were obtained from studies where amniotic fluid samples were withdrawn either by amniocentesis, from uncontaminated samples during labor, or at birth during scheduled cesarean sections. The weighted mean fetal creatinine levels showed a steady increase with gestational age, from 3.2 mg/L at 9 weeks GA to 25.7 mg/L at term.

**Table 2:** Summary of studies with measured values of amniotic fluid creatinine concentration (mg/L) at different gestational ages.

**Reference**	**Method of amniotic fluid sample collection**	**Number of subjects**	**Gestational age (weeks)**	**Creatinine conc (Mean ± SD)**	**Sample analysis method**
Gulbis et al. (32)	Transabdominal amniocentesis	59 healthy fetuses	8–16	29–63 (range)	Jaffe Picric acid reaction
Oliveira et al. (33)	Amniocentesis	115	13–20 27–34 36–42	0.6 ± 0.07 1.28 ± 0.34 1.83 ± 0.42	Mega Kits Merck Diagnostic
Droegemueller et al. (34)	Amniocentesis	65	29–41	10–25 (range)	Jaffe picric acid method with an autoanalyzer
Jauniaux et al. (35)	Transvaginal guided amniocentesis	17	5–13	3.13 ± 0.67	Commercial kit (Boehringer)
Campbell et al. (36)	Transvaginal guided amniocentesis	40	7–12	4.2 ± 1.4	Merck ERIS multichannel analyser
Jauniaux et al. (37)	Transvaginal guided amniocentesis	32	11–14 12–16	4 ± 1 7 ± 1	Commercial kit (Boehringer)
Benzie et al. (38)	Amniocentesis, Amniotomy and Hysterotomy	208	15 19 22 25 28 31 34 37 40	7 ± 1 7 ± 1 6 ± 1 7 ± 1 11 ± 3 14 ± 3 17 ± 4 21 ± 5 22 ± 5	Jaffe reaction (modification using the Techno analyser method)
Doran et al. (39)	Amniocentesis	131	13–>35	9.7–18.8	Jaffe picric acid method with an autoanalyzer
Pitkin and Zwirek (40)	Transabdominal amniocentesis	119	21–42	8.4–31.8 (range)	Jaffe Picric acid reaction
Emara et al. (41)	Transabdominal and transvaginal amniocentesis	42	40	21.5 ± 4.4	Jaffe reaction colorimetric method
Fex et al. (42)	Abdominal amniocentesis	189	15–43	5.8–45.2 (range)	Jaffe's method
Troccoli et al. (43)	Amniocentesis	29	16 17 18 19 20	9.54 ± 1.35 9.26 ± 1.95 10.8 9.32 ± 0.64 9.7	Jaffe method colorimetric test
Tzschoppe et al. (44)	Amniocentesis	200	16 16	7.1 ± 0.4 6.2 ± 0.2	Creatinine enzymatic reaction
Burghard et al. (45)	Abdominal amniocentesis and vaginally	171	16–17 18–19 20–23 24–27 28–32 33–36 37–38	6.9 (3.3–9.5) 6.9 (5.2–12) 7.6 (5.1–8.5) 8 (7.1–9) 11.4 (4.1–14.5) 13.1 (5.1–22.8) 15.9 (12.8–19.9) Mean (Range)	Jaffe Picric acid reaction
Mussap et al. (46)	Amniocentesis	55	15–21 22–36 37–40	6.6 ± 0.3 10.6 ± 2.3 19.5 ± 3.4	Kinetic Jaffe Picric acid reaction
Lind et al. (47)	Abdominal amniocentesis	219	6–20 <3 31- 33- 35- 37- >39	5.7 ± 1.5 8 ± 1.8 12 ± 2.4 13 ± 2.6 15 ± 2.8 19 ± 5 20 ± 4	Jaffe precipitation reaction

**Figure 3:**
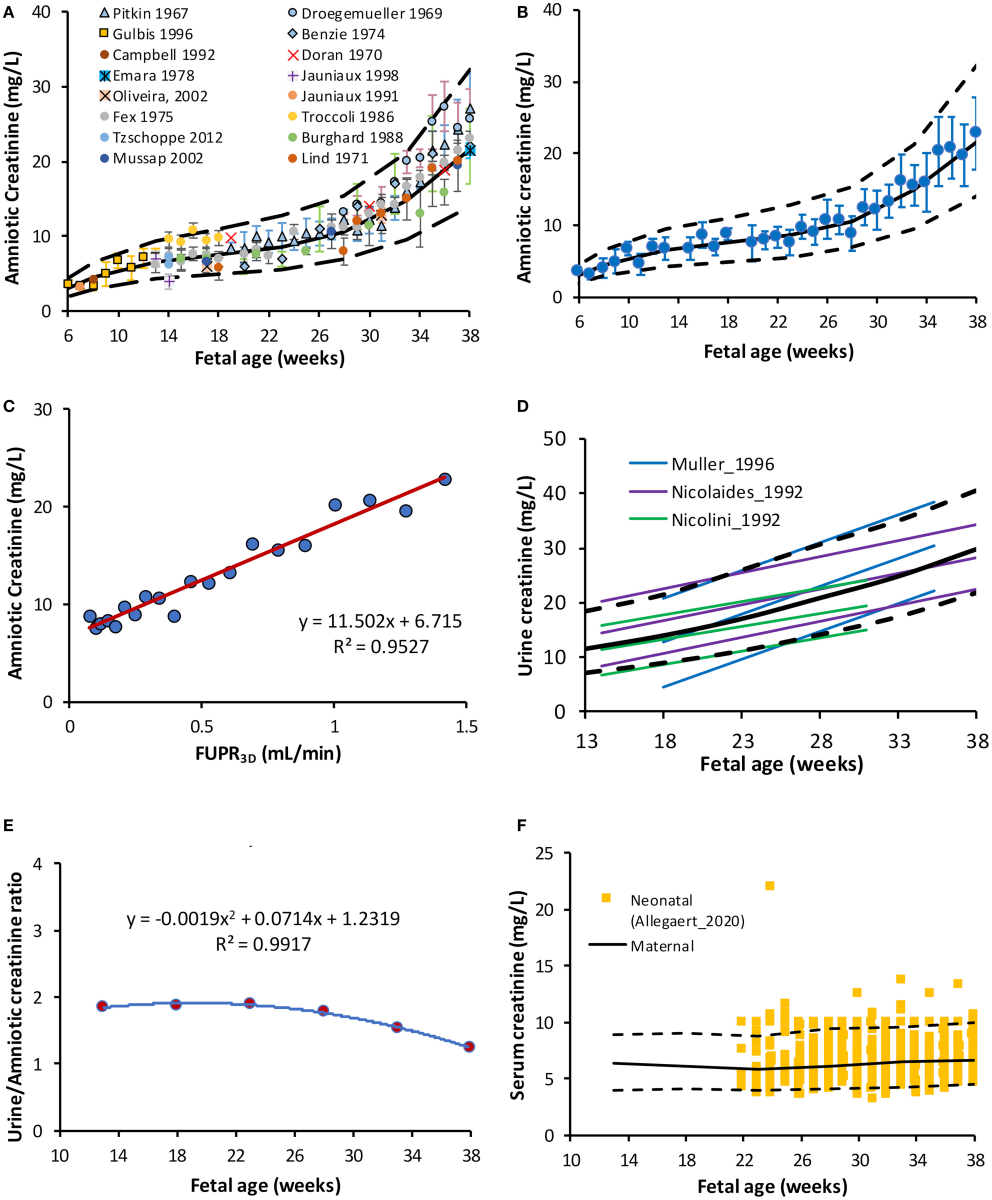
Creatinine level in the amniotic and fetal fluids. **(A)** Fetal amniotic creatinine concentration as a function of fetal age, **(B)** weighted means (circles) and SD of amniotic creatinine level plotted against Fetal age, **(C)** Amniotic creatinine concentration against predicted FUPR based on the function derived from 3D data **(D)** reported references values for the creatinine in fetal urine from fetuses diagnosed with (6, 48) and without (49) urinary tract uropathies **(E)** Fetal amniotic to urine creatinine ratio, and **(F)** Serum creatinine in maternal serum [lines (50)] overlaying measured neonatal serum creatinine at birth [squares (51)]. Solid and broken lines represent the median and 5th-95th intervals.

Collected data on changes of amniotic creatinine concentration indicate a rapid increase in amniotic fluid creatinine concentration (mg/L) from a mean (SD) of 4.5 (1.1) at 10 GAs to 7.5 (1.8) at the middle of pregnancy reaching a value of 22 (5.4) at term (Figures 3A,B). The longitudinal changes in measured amniotic fluid concentration with gestational age between 5 and 40 GAs was best described by a 3rd order polynomial function (*R*^2^ = 0.97):


(8) $$\begin{array}{r} Amniotic\;Creatinine\;\left(mg/L\right)=0.0012\;{FA\;}^{3}-0.0632\;{FA\;}^{2}\\ +1.304\;FA-2.4653 \end{array}$$

(9) $$\begin{array}{r} Amniotic\;Creatinine\;\left(mg/L\right)=0.0012\;{GA\;}^{3}-0.0705\;{GA\;}^{2}\\ +1.5714\;GA-5.3358 \end{array}$$

A plot of predicted amniotic creatinine concentration vs. the predicted FUPR at different fetal ages indicate a strong linear relationship (*R*^2^ = 0.9527) between these two variables (Figure 3C).

### Fetal Urine Creatinine

Information on changes in fetal UrCr were limited to three studies (6, 48, 49), where samples were obtained from fetal renal pelvis, ureter, or bladder (Supplementary Table 3). The following polynomial function was derived to describe these three studies:


(10) $$\begin{array}{l} Fetal\;UrCr\;\left(mg/L\right)=\;8.43+0.0720\;GA\;+0.0119\;{GA}^{2} \end{array}$$

(11) $$\begin{array}{l} Fetal\;UrCr\;\left(mg/L\right)=\;8.62+0.1195\;FA\;+0.0119\;{FA}^{2} \end{array}$$

This equation was used to calculate fetal GFR (Scenario A). Data from two studies (6, 48) were obtained from fetuses with different uropathies, but were either “normal” with respect to their postnatal function or their kidney, which were histologically normal before elective termination of pregnancy, The fetal GFR was also assessed using the original equation reported by Nicolini et al. (49) for their “normal” fetuses aged 16–33 GAs:


(12) $$\begin{array}{l} Fetal\;UrCr\;\left(mg/L\right)=\;\left(31.2+4.29\;GA\right)\left(\frac{10}{88.42}\right) \end{array}$$

The term (10/88.42) is for unit conversion from μmol/L to mg/L. Since this equation was derived from fetal data between 16 and 33 GAs, an assumption was made that this equation can be used to predict fetal UrCr at term (Scenario B).

### Fetal Serum Creatinine

Data search retrieved limited information on fetal SerCr concentration (Supplementary Table 4). Fetal SerCr was shown to be similar to maternal level in 63 pregnancies at 20-26 GAs with fetal/maternal ratio =1 (52). Likewise, in another study with a cohort of mothers and their fetuses (*n* = 522), serum samples were simultaneously measured, and the maternal and fetal SerCr levels showed an equilibrium (fetal/maternal ratio = 1) from 16 GAs until term (53). Unfortunately, neither the fetal nor the maternal SerCr was reported separately. Previously, we reported an equation based on the meta-analysis of the maternal SerCr at different gestational weeks (*R*^2^ = 0.9543) (50):


(13) $$\begin{array}{r} Maternal\;SerCr\;\left(mg/L\right)=10\;(0.80-0.0147\;GA\\ +0.0003\;{GA}^{2}) \end{array}$$

Data were generated using this function assuming 25% CV and then compared against 4,509 SerCr measurements obtained at birth (23–42 GAs) from 1,181 newborns (51). Due to its low molecular weight (113.12 g/mol), creatinine freely passes the placental barrier and its level at birth is known to reflect the maternal level. Indeed, the comparison of maternal SerCr and neonatal SerCr results showed perfect overlap (Figure 3F) indicating the adequacy of using maternal SerCr for calculating the fetal GFR (i.e., *Fetal SerCr* = *Maternal SerCr* = *neonatal SerCr at birth*).

### Fetal GFR

**Figure 4:**
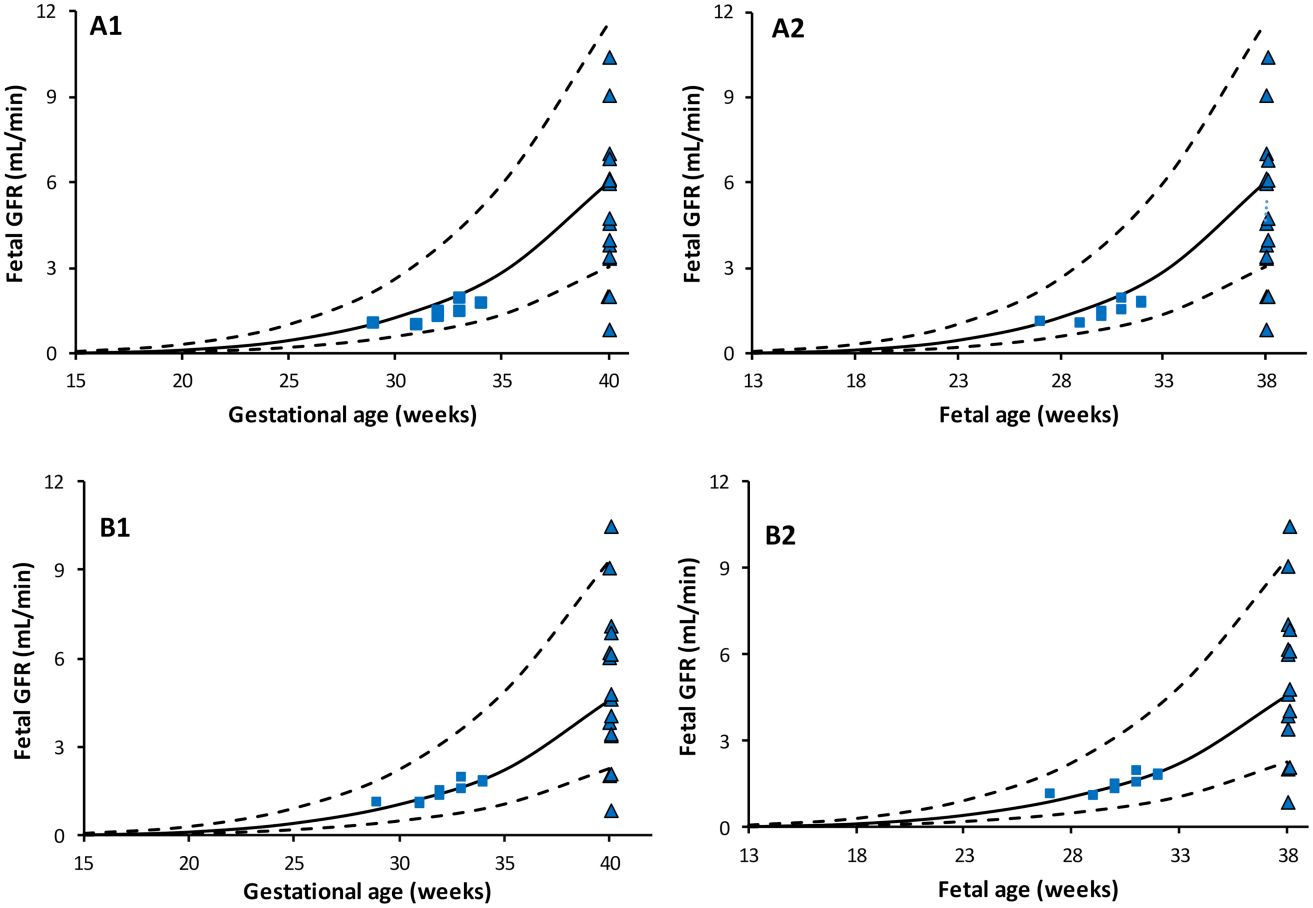
Predicted Fetal GFR profiles at different gestational (left) and fetal (right) ages. Scenario **(A)** using maternal SerCr in place of fetal SerCr, while fetal UrCr used equation based on data that include normal fetuses (49) and fetuses diagnosed with uropathies (6, 48). Scenario **(B)** using maternal SerCr in place of fetal SerCr, while fetal UrCr is only based on “normal” fetuses as per Nicolini et al. Solid lines represent median, broken lines represent 5th and 95th percentiles. Shapes represent observed neonatal GFR measured within few hours after birth in full term [triangle, Strauss et al. (54)] and preterm [squares: Coulthard et al. (55)] subjects.

Predicted fetal GFR values at different gestational weeks are given in Table 3 for the different fetal urine creatinine assumptions. Plot of the predicted fetal GFR profiles at different gestational and fetal ages are given in Figure 4. The profiles show good agreement with neonatal GFR values measured for preterm and term neonates within the first postnatal day.

**Table 3:** Predicted values for fetal urine production rate (FUPR), creatinine level and GFR at different gestational weeks.

	**Gestational Week**	**10 weeks**	**15 weeks**	**20 weeks**	**25 weeks**	**30 weeks**	**35 weeks**	**40 weeks**
FUPR_3D_ (ml/min)	Mean ± SD	NA	0.016 ± 0.005	0.062 ± 0.018	0.176 ± 0.054	0.40 ± 0.122	0.80 ± 0.23	1.42 ± 0.42
	Median (5th-95th percentile)	NA	0.015 (0.009–0.024)	0.060 (0.038–0.093)	0.168 (0.102–0.28)	0.38 (0.24–0.62)	0.77 (0.48–1.22)	1.354 (0.85–2.24)
Amniotic Creatinine (mg/L)	Mean ± SD	4.54 ± 1.10	6.47 ± 1.56	7.52 ± 1.84	8.63 ± 2.14	10.6 ± 2.6	14.9 ± 3.7	21.6 ± 5.4
	Median (5th-95th percentile)	4.54 (2.72–6.27)	6.4 (4.3–9.4)	7.3 (4.97–10.98)	8.3 (5.60–12.85)	10.3 (7.0–15.4)	14.6 (9.5–21.2)	20.6 (14.0–32.4)
Fetal SerCr (mg/L)	Mean ± SD	NA	6.39 ± 1.54	6.23 ± 1.54	6.07 ± 1.5	6.36 ± 1.62	6.65 ± 1.6	6.89 ± 1.64
	Median (5th-95th percentile)	NA	6.3 (4.0–8.9)	6.1 (4.1–9.0)	5.9 (4.0–8.8)	6.2 (4.1–9.5)	6.5 (4.3–9.6)	6.7 (4.5–10)
Fetal UrCr (mg/L)^**^	Mean ± SD	NA	12.0 ± 3.6	14.4 ± 4.0	17.5 ± 4.6	21.3 ± 5.1	25.2 ± 5.5	30.2 ± 5.7
	Median (5th-95th percentile)	NA	11.4 (7.0–18.5)	13.9 (8.9–21.5)	16.9 (11.1–26.0)	20.8 (14.1–30.7)	24.7 (17.4–35.1)	29.7 (21.8–40.6)
GFR (ml/min) (Scenario A)	Mean ± SD	NA	0.03 ± 0.02	0.15 ± 0.08	0.5 ± 0.3	1.4 ± 0.7	3.2 ± 1.5	6.5 ± 2.7
	Median (5th-95th percentile)	NA	0.03 (0.01–0.06)	0.14 (0.06–0.31)	0.48 (0.22–1.0)	1.3 (0.60–2.6)	2.9 (1.4–6.0)	6.0 (3.1–11.6)
GFR (ml/min) (Scenario B)	Mean ± SD	NA	0.03 ± 0.01	0.14 ± 0.07	0.47 ± 0.24	1.20 ± 0.57	2.55 ± 1.29	4.92 ± 2.12
	Median (5th-95th percentile)	NA	0.02 (0.01–0.05)	0.12 (0.06–0.28)	0.43 (0.20–0.91)	1.08 (0.50–2.26)	2.24 (1.07–4.91)	4.49 (2.23–9.14)

*FUPR_3D_, fetal urine production rate for the 3D measurement techniques; SerCr, serum creatinine; UrCr, urine creatinine; NA, not calculated/not applicable*.

Assuming 30%CV for FUPR and 25% for amniotic creatinine as well as for SerCr, and age dependent (%CV= −0.4*FA + 35.2) for UrCr.

** *For all urine data, including fetuses diagnosed with uropathies. See fetal urine creatinine section for details on the different GFR scenarios*.

## Discussion

This study quantifies the longitudinal increase of FUPR with respect to fetal age and the associated changes in creatinine levels in the fetal urine and amniotic fluid in uncomplicated pregnancies. These findings have potential applications in the assessment of normal fetal GFR maturation. Different studies have previously measured FUPR as an assessment of fetal renal function and perinatal outcomes in both normal and complicated pregnancies (21, 22, 30). FUPR estimation depends on the volume of the bladder, which is neither regular nor static, due to filling and emptying activities during fetal micturition. Historically, the 2D ultrasonic measurements derive bladder volume using an assumption that the bladder has a static ovoid or ellipsoid shape to compensate for the lack of depth information (27). The ellipsoid formula may underestimate or overestimate the bladder volume by up to 33%, depending on what bladder shape was being considered (56). In addition, since the mean time of the fetal bladder cycle is about 25 min (23), an estimated FUPR will also depend on the interval of bladder volume measurements. Studies conducted using real ultrasounds were able to capture the activity of the fetal bladder, and more frequent measurements at about 5 min intervals, produced higher FUPR than those made at 15-to-30-min intervals (Table 1).

The introduction of 3D ultrasonography offers more accurate volume measurements of irregular shapes (including fetal bladder volume) compared to the 2D ultrasonography, including fetal bladder volume as it quantifies the volume from the three spatial dimensions. When 3D ultrasonography is coupled with the rotational multiplanar technique VOCAL, a detailed and quick acquisition of the bladder volume can be obtained with lower inter- and intra-observer variability (57) and more reliable FUPR estimation than those measured using the 2D or manual planimetry 3D ultrasound (58).

The current study utilized FUPR values measured using 2D or 3D ultrasound to derive a function to predict changes of FUPR with fetal age. Previous studies derived various functions (Table 1) and proposed either a linear or a second order polynomial function (Table 1). Only one study proposed a power model function (20). From our analysis, the polynomial regression for the 3D data had the highest R^2^ value indicating the best fit to the observed data, however, it predicted negative values for FUPR before the 19th fetal week (Supplementary Figure 1). Since it is known that fetal urine production begins around 13 weeks, this function was not selected. Given that the difference between MSE and R^∧^2 for the power law and the polynomial regressions are small (Supplementary Table 1), the power law function was chosen for describing FUPR. The FUPR_2D_ predicted a maximum fetal urinary flow rate of 0.5 ml/min (0.03 L/h) at 40 weeks GA. This value is ~3-fold less than the mean estimated value of 1.41 ml/min (0.085 L/h) at term using the FUPR_3D_ technique. A correction function for the FUPR_2D_ data has therefore been derived to facilitate FUPR measurement conversion at different fetal or gestational ages.

Assessment of fetal renal maturation has been attempted by different methods, including the measurement of amniotic fluid volume, clearance of endogenous or exogenous filtration markers, urine production and urinary analyte levels, which can be measured in either fetal urine or in amniotic fluid (6, 15). The volume of amniotic fluid is a poor indicator of renal function since it is not determined by the fetus only, but also by the mother, especially during the first half of pregnancy. Fetal urine becomes a major contributor to the amniotic fluid volume during the second half of pregnancy, and its contribution increases with pregnancy progression until term. The concentration of urinary analyte levels in the amniotic fluid is also challenged by identifying a pure “urinary analyte” that is of a large enough molecular weight (MWt), that it does not cross the maternal-amniotic and fetal-amniotic barrier by any route, but small enough to pass through the fetal glomerular pores by simple filtration and excreted into the amniotic fluid.

Creatinine (MWt of 113 g/mol) is commonly used for assessing postnatal renal function. Its amniotic fluid levels increase rapidly between 8 and 16 GAs, whilst its level in maternal serum remains almost unchanged, which indicates increasing excretion by the fetus into the amniotic fluid (32). Collected data indicated that the creatinine level in the amniotic fluid increase during pregnancy at different rates during the different gestational periods (Figures 3A,B). This change was strongly correlated with FUPR (*R*^2^ = 0.95) implying the role of fetal urinary contribution.

Collected data on fetal UrCr at different fetal ages (Supplementary Table 3) were limited to three studies (6, 48, 49), but all the studies showed a linear increase with progression of the gestation. While the rate of increase in the fetal UrCr was constant in each study, the rate was different between these studies as shown in Figure 3D. This is probably because the fetuses had different underlying uro-pathological disorders as they were diagnosed with (bilateral) uropathies before urine collection, even though they were reported to be normal with respect to postnatal renal function or have no histological signs of renal dysplasia. The relationship between the fetal UrCr and amniotic creatinine was linear, with UrCr levels almost twice the amniotic creatinine until near term, where creatinine concentration in the amniotic fluid starts to rise faster than fetal UrCr (Figure 3E). This is probably explained by the fact that the amniotic fluid volume is decreasing toward term (50) at a faster rate than the maturation of the glomerulus filtration.

Collected data on the fetal SerCr indicated that the levels of fetal SerCr is lower than the levels of fetal UrCr or the Amniotic creatinine (Supplementary Table 4) but showed a continuous increase during development. This increase in the fetal SerCr level reflects the maternal SerCr during the gestational period, since creatinine passes freely between the mother and the fetus through the placenta. In a cohort of pairs of mothers and fetuses (*n* = 522), SerCr levels were measured simultaneously and it was found that both maternal and fetal SerCr level were in equilibrium from 16 GAs until term in one study (53) and between 20 and 26 GAs in another (52). The maternal SerCr was reported to increase toward term from a nadir level recorded at the midpoint of pregnancy, but still below the non-pregnant level (59, 60).

On the other hand, one study that compared the fetal and maternal plasma levels of creatinine longitudinally from 15 GAs until birth reported a gestational-dependent decrease in maternal plasma creatinine, whilst showing continuous increase in the fetal SerCr during pregnancy that crossed over the maternal levels near term (61). This study however reported lower maternal and fetal SerCr creatinine levels across the whole gestational period compared with other studies (59, 60, 62, 63). Similar observation of an increase in fetal SerCr relative to maternal SerCr near term was reported based on limited samples (64).

In the current work, since fetal SerCr is a required parameter for the fetal GFR calculation, (see Method section), we have compared the maternal SerCr using a previously developed gestational age-dependent maternal SerCr function (50) with neonatal SerCr obtained at birth from 1,181 newborns between 23 and 42 GAs (51) to assess any trend. As shown in Figure 3F, the neonatal data were almost identical to the maternal SerCr at different weeks of pregnancy, therefore the maternal data was used, rather than the neonatal data since the original function was derived from observed data during the different trimesters, while extrapolating the neonatal data to GAs earlier than 24 GAs is questionable.

The calculated fetal GFR based on FUPR, and creatinine data are given in Figure 4, which also shows reported data determined immediately after birth in healthy preterm (55) and term (54) neonates. Since two fetal UrCr data sets stemmed from cases diagnosed with fetal uropathies, other than dysplasia, two scenarios were assessed; if the three studies were combined (Figure 4A), or if only data from “normal” fetuses (49) were used (Figure 4B). In general, there is a slight over estimation of neonatal GFR at birth, especially when fetal UrCr data from the combined data set was used. Fetal GFR at term may not reflect the neonatal at birth due to the observed rapid drop in the neonatal renal vascular resistance and the increase in the renal blood flow (17, 18) unless these changes are associated with reduction in the renal UrCr. This is because the neonatal SerCr at birth is still similar to fetal SerCr at term. The interpretation of these prediction should be in the context of the current knowledge on the change in the underlying physiological parameters and further assessments are required.

Quantifying the maturation of fetal renal function has its place in PBPK modeling, especially for renally cleared compounds after maternal intake such as most anti-infective drugs including gentamicin, amikacin, penicillin, cephalosporin, tenofovir after maternal intake. Given the difficulty in measuring fetal GFR, quantification of an alternative method can facilitate PK assessment of drug exposure within the feto-placental unit during pregnancy. Providing a continuous equation to describe the fetal renal maturation process also increases the applicability of PBPK at different weeks of fetal age.

Finally, this work has different limitations. This work quantifies the increase in the fetal renal function, based on changes in FUPR obtained from different studies carried out in different ethnic groups to construct the observed changes during fetal development. The impact of ethnicity, if it exists, was not considered due to data limitation. However, the overlap of FUPR measurements in Asians and Caucasians (Table 1) suggests that ethnicity may not influence FUPR. The FUPR function was derived based on data available from 18 GAs onwards and extrapolating the results to earlier ages should be carefully assessed.

Another limitation is that some studies reported results as summary statistics, while others reported individual values. Integration of these data were performed after calculating summary statistics from individual data, which can result in an under-estimation of interindividual variability of FUPR. Fetal and maternal covariates were not available to enable inclusion of physiological covariates in the FUPR functions.

Finally, it is not known to what degree the fetal FUPR is affected by maternal illness, for example maternal renal impairment. Therefore, in this work, only data from healthy pregnancies were selected. This is because the understanding of normal fetal renal physiology is a prerequisite when quantifying the impact of different disease states on fetal renal function. Some of the studies which we used in our analysis compared FUPR in healthy pregnancies with FUPR in medically complicated pregnancies, and showed that FUPR is significantly different only when the pregnancy complication affects the growth of the fetus (21, 30). Examples of medical conditions affecting fetal urine production are provided in the Supplementary Table 5.

In addition, we did not attempt to assess other renal function biomarkers or amniotic fluid composition during fetal development. The amniotic level of β2-microglobulin increases from 7.9 (1.0–24.7) mg/L at 16–17 GAs to 11.6 (3.4–14.1) mg/L at 24–27 GAs (45) indicating an increase in the fetal synthesis together with the maturation of renal filtration (fetal serum: 3.4 (2.0-4.9) mg/L between 18 and 38 GAs (65) vs. mean maternal serum ranged from 1.45 to 1.7 mg/L at 7–40 GAs (66). During the second trimester, the amniotic β2-microglobulin level declines to reach about 2.4 (1.0–6.1) mg/L at term (45). This reduction may indicate maturation of both the reabsorption and degradation of β2-microglobulin in the proximal tubules (32). However, the level of β2-microglobulin is sensitive to a triggered immune response and it has been shown to be elevated in fetuses with uropathy (67). The application of any of these renal function biomarkers, separately or in combinations, has advantages in assessing fetal renal function. However, dynamic modeling of their excretion simultaneously with maternal, fetal, and amniotic levels form longitudinal studies is required to quantify a reliable GFR marker.

In conclusion, age-dependent functions for FUPR, amniotic creatinine, and fetal urine creatinine have been derived in this study by integrating data from multiple studies to predict fetal urine flow during development and can be used for assessing the fetal GFR at different fetal ages. The absence of fetal GFR measurements, due to ethical reasons, challenges the verification of the predicted values. A possible way to assess the suitability of the presented approach of predicting fetal GFR is by integrating it within a fetal PBPK model to predict fetal and amniotic exposure of renally eliminated compounds at different GAs. The fetal GFR results reflect the current knowledge and data availability. Additional studies are required to establish reference values for normal fetal urine and serum creatinine levels from sufficient subjects at different gestational weeks to build the confidence in the normal fetuses as a reference population so the revision of the fetal GFR can be continued and the impact of diseases on fetal renal function mechanisms can be quantified.

## Data Availability Statement

The original contributions presented in the study are included in the article/Supplementary Material, further inquiries can be directed to the corresponding author.

## Author Contributions

UE, AP, and KA collected the data. KA, UE, AP, and AB analyzed the data. UE, AB, and KA wrote the manuscript. All authors reviewed the manuscript and contributed to the article and approved the submitted version.

## Conflict of Interest

All authors are paid employees of Certara UK Limited (Simcyp Division) and may hold shares in Certara.

## Publisher's Note

All claims expressed in this article are solely those of the authors and do not necessarily represent those of their affiliated organizations, or those of the publisher, the editors and the reviewers. Any product that may be evaluated in this article, or claim that may be made by its manufacturer, is not guaranteed or endorsed by the publisher.
