# Supplementary material for: Quantification of Fetal Renal Function Using Fetal Urine Production Rate and Its Reflection on the Amniotic and Fetal Creatinine Levels During Pregnancy
Source: Front Pediatr. 2022 Mar 3;10:841495. doi: 10.3389/fped.2022.841495 (PMC8927781; doi:10.3389/fped.2022.841495)
Supplement: Supplementary file 1 [file Data_Sheet_1.docx]

Quantification of Fetal Renal Function using Fetal Urine Production Rate and its Reflection on the Amniotic and Fetal Creatinine levels During Pregnancy

Udoamaka Ezuruike^1^, Alexander Blenkinsop^1^, Amita Pansari^1^, Khaled Abduljalil^1*^

^1^Certara UK Limited (Simcyp Division), Level 2-Acero, 1 Concourse Way, Sheffield, S1 2BJ, United Kingdom

***Correspondence:**Khaled Abduljalil

Khaled.abduljalil@certara.com

Keywords: Fetus, Pregnancy, Urine Production, Renal function, Creatinine, PBPK.

Figure S1: 3D (A) ultrasound data and 2D (B) data fitted to different functions.

Table 1: Functions with parameters fitted to 3D ultrasound data

| **Function type** | **Function** | **Parameters** | | | **Weighted Mean Squared Error (×10^-4^)** | **R^2^** |
| --- | --- | --- | --- | --- | --- | --- |
|  |  | **A** | **B** | **C** |  |  |
| Linear | $FUPR=A . FA + B$ | 0.069 | -1.428 |  | 7.65 | 0.972 |
| Exponential | $FUPR=A . e^{(B . FA)}$ | 0.00663 | 0.144 |  | 9.81 | 0.943 |
| Power Law | $FUPR=A . {FA}^{B}$ | 3.21×10^-7^ | 4.207 |  | 8.18 | 0.953 |
| Polynomial | $FUPR=B . FA+A \cdot{FA}^{2}+ C$ | -0.01472 | -0.03 | 0.001674 | 7.06 | 0.977 |

FA: Fetal age in weeks (= Gestational week – 2 weeks)

Table 2: Functions with parameters fitted to 2D ultrasound data

| **Function type** | **Function** | **Parameters** | | | **Weighted Mean Squared Error (×10^-4^)** | **R^2^** |
| --- | --- | --- | --- | --- | --- | --- |
|  |  | **A** | **B** | **C** |  |  |
| Linear | $FUPR=A . FA + B$ | 0.018 | -0.236 |  | 1.2 | 0.985 |
| Exponential | $FUPR=A . e^{(B . FA)}$ | 0.0297 | 0.074 |  | 2.22 | 0.973 |
| Power Law | $FUPR=A . {FA}^{B}$ | 1.69×10^-4^ | 2.187 |  | 1.67 | 0.981 |
| Polynomial | $FUPR=B . FA+A \cdot{FA}^{2}+ C$ | -0.696 | 0.0507 | 0.001674 | 0.97 | 0.987 |

**Table 3.** Creatinine level in the fetal urine during development (mg/dL)

| **GW (n)** | **Mean SD** |  | **comments** | **Reference** |
| --- | --- | --- | --- | --- |
| Fetal urine |  |  |  |  |
| 16  18  20  22  24  26  28  30  32  33 | 1.13±0.45  1.23±0.45  1.32±0.45  1.42±0.45  1.52±0.45  1.61±0.45  1.71±0.45  1.81±0.45  1.91±0.45  1.95±0.45 |  | Total of 26 fetuses without urinary tract abnormalities | (Nicolini et al., 1992)  Data generated for the studied range using the reported function  Fetal urinary creatinine (umol/L) = 31.2+4.29*GW and  SD=39.5 umol/L |
| 16  20  24  28  32  36  40 | 1.44±0.59  1.67±0.59  1.90±0.59  2.13±0.59  2.36±0.59  2.59±0.59  2.83±0.59 |  | Total of 20 fetuses (13 alive neonates with normal renal function and 6 died (1 in utero and 5 electively terminated pregnancy) had no histological evidence of renal dysplasia | (Nicolaides et al., 1992) |
| 20  22  24  26  28  30  32  34  36  37 | 1.27±0.91  1.48±0.91  1.69±0.91  1.88±0.91  2.09±0.91  2.29±0.91  2.49±0.91  2.71±0.91  2.89±0.91  2.98±0.91 |  | Total of 39 fetuses with uropathy but without urinary tract abnormalities | (Muller et al., 1996) |

**Table 4.** Creatinine level in the fetal serum during development (mg/L)

| **GW (n)** | **Mean**±**SD** | Assay | **comments** | **Reference** |
| --- | --- | --- | --- | --- |
| 15-18 (79)  19-22 (38)  23-26 (18)  27-30 (22)  31-34 (25)  35-38 (162) | 4.07 (3.39-5.32)  4.64 (3.62-5.88)  4.75 (3.73-5.65)  4.75 (3.85-5.65)  4.86 (3.62-6.11)  5.32 (3.62-7.35) | Jaffe alkaline picrate | Mean (range) | (Moniz et al., 1985) |
| 20 -26 | 7.3±0.2 | (modified) Jaffe method | Total n=63 fetuses,  No difference from maternal | (Forestier et al., 1987) |
| 12-16 (12) | 4.0±1.0 | Enzymatic |  | (Jauniaux et al., 1998) |
| During delivery (27) | 6.8±1.4 | Enzymatic | Umbilical artery level is not different from the umbilical vein | (Kristensen et al., 2008) |

Table 5. Example of medical conditions affecting urine production

| Condition | Observed effect on FUPR | Reference |
| --- | --- | --- |
| Bilateral renal agenesis | 100% reduction at 21-23 weeks | (Takeuchi et al., 1994) |
| Bilateral infantile polycystic kidney | 100% reduction at 21-28 weeks | (Takeuchi et al., 1994) |
| Twin transfusion syndrome (TTS) | 100% reduction at 21-28 weeks | (Takeuchi et al., 1994) |
| Bilateral hydronephrosis | 60% reduction at 21-38 weeks | (Takeuchi et al., 1994) |
| Isolated oligohydramnios (n = 34) | FUPR was not decreased at term. | (Seol et al., 2021) |
| IUGR  (n = 17) | Decreased FUPR in patients with IUGR at term. | (Seol et al., 2021) |
| Congestive heart failure  (n = 11) | Fetuses with CHF had decreased UPR values at 21 to 40 GWs | (Xie et al., 2012) |
| (unexplained) polyhydramnios  (n=5) | Higher FUPR at 25-40 GWs | (Touboul et al., 2009) |
| Non-controlled gestational diabetes (n=30) | Higher FUPR at 24–37 GWs | (Maged et al., 2014) |
| Uteroplacental insufficiency with adverse pregnancy outcomes | 42% reduction of FUPR toward the end of the pregnancy | (Lee et al., 2014) |

**References**

Forestier, F., Daffos, F., Rainaut, M., Bruneau, M., and Trivin, F. (1987). Blood chemistry of normal human fetuses at midtrimester of pregnancy. *Pediatr Res* 21(6)**,** 579-583. doi: 10.1203/00006450-198706000-00015.

Jauniaux, E., Gulbis, B., Hyett, J., and Nicolaides, K.H. (1998). Biochemical analyses of mesenchymal fluid in early pregnancy. *Am J Obstet Gynecol* 178(4)**,** 765-769. doi: 10.1016/s0002-9378(98)70489-2.

Kristensen, K., Strevens, H., Lindstrom, V., Grubb, A., and Wide-Swensson, D. (2008). Increased plasma levels of beta2-microglobulin, cystatin C and beta-trace protein in term pregnancy are not due to utero-placental production. *Scand J Clin Lab Invest* 68(7)**,** 649-653. doi: 10.1080/00365510802007804.

Lee, S.M., Jun, J.K., Kim, S.A., Lee, E.J., Kim, B.J., Park, C.W., et al. (2014). Usefulness of fetal urine production measurement for prediction of perinatal outcomes in uteroplacental insufficiency. *J Ultrasound Med* 33(12)**,** 2165-2171. doi: 10.7863/ultra.33.12.2165.

Maged, A.M., Abdelmoneim, A., Said, W., and Mostafa, W.A. (2014). Measuring the rate of fetal urine production using three-dimensional ultrasound during normal pregnancy and pregnancy-associated diabetes. *J Matern Fetal Neonatal Med* 27(17)**,** 1790-1794. doi: 10.3109/14767058.2013.879709.

Moniz, C.F., Nicolaides, K.H., Bamforth, F.J., and Rodeck, C.H. (1985). Normal reference ranges for biochemical substances relating to renal, hepatic, and bone function in fetal and maternal plasma throughout pregnancy. *J Clin Pathol* 38(4)**,** 468-472. doi: 10.1136/jcp.38.4.468.

Muller, F., Dommergues, M., Bussieres, L., Lortat-Jacob, S., Loirat, C., Oury, J.F., et al. (1996). Development of human renal function: reference intervals for 10 biochemical markers in fetal urine. *Clin Chem* 42(11)**,** 1855-1860.

Nicolaides, K.H., Cheng, H.H., Snijders, R.J., and Moniz, C.F. (1992). Fetal urine biochemistry in the assessment of obstructive uropathy. *Am J Obstet Gynecol* 166(3)**,** 932-937. doi: 10.1016/0002-9378(92)91367-j.

Nicolini, U., Fisk, N.M., Rodeck, C.H., and Beacham, J. (1992). Fetal urine biochemistry: an index of renal maturation and dysfunction. *Br J Obstet Gynaecol* 99(1)**,** 46-50. doi: 10.1111/j.1471-0528.1992.tb14391.x.

Seol, H.J., Kim, H.Y., Cho, G.J., and Oh, M.J. (2021). Hourly fetal urine production rate in isolated oligohydramnios at term. *PLoS One* 16(5)**,** e0250659. doi: 10.1371/journal.pone.0250659.

Takeuchi, H., Koyanagi, T., Yoshizato, T., Takashima, T., Satoh, S., and Nakano, H. (1994). Fetal urine production at different gestational ages: correlation to various compromised fetuses in utero. *Early Hum Dev* 40(1)**,** 1-11. doi: 10.1016/0378-3782(94)90094-9.

Touboul, C., Picone, O., Levaillant, J.M., Boithias, C., Frydman, R., Boulvain, M., et al. (2009). Clinical application of fetal urine production rate in unexplained polyhydramnios. *Ultrasound Obstet Gynecol* 34(5)**,** 521-525. doi: 10.1002/uog.6440.

Xie, J.X., Lv, G.R., Chen, Q.Y., and Hou, M. (2012). The effect of fetal congenital heart disease on in utero urine production rate. *Prenat Diagn* 32(6)**,** 536-541. doi: 10.1002/pd.3852.
